# From Identity to Enaction: Identity Behavior Theory

**DOI:** 10.3389/fpsyg.2021.679490

**Published:** 2021-08-24

**Authors:** Jack D. Simons

**Affiliations:** Department of Counseling, Mercy College, Dobbs Ferry, NY, United States

**Keywords:** action theory, attitudes, Identity Behavior Theory, minority, resilience, intention, personal strength, social support

## Abstract

The article deals with the meaning of identity in action regulation. A strengths-based action model, Identity Behavior Theory (IBT), is concerned with the role that identity plays in the prediction of behavioral enaction, and implications for education, science, and clinical practice. With this respect the article explores and discusses how enacted behavior, including intention and action, depends on level of subscription to identity as well as on resilience and attitudes that are related to such a behavior. The article also illustrates fields of application of IBT, use of IBT with underrepresented and marginalized groups, and as an instrument for assessing and testing possible effects of resilience, attitudes, and identity on the enacted behavior. IBT is now used to examine behavior in a variety of educational contexts in the United States, and more studies are needed to satisfactorily validate application of the model empirically.

## Introduction

We need to be alert for concepts and methods that enable us to understand individuality. -Allport

Identity Behavior Theory (IBT) is a strengths-based action model of behavior concerned with the role that identity plays in the prediction of behavioral enaction, the process whereby individuals shape their experiences through planning and successful action. Behavioral enaction comprises behavioral intention and behavioral action, and, as part of IBT, is assessed along with identity, attitudes, and resilience, the latter of which comprises personal strength (e.g., emotion regulation and self-esteem) and support (e.g., having positive role models). IBT is assumed to be valid for use with the population at large but even more so valid for use with marginalized people who need empowering processes to act. Thus, on one hand IBT is assumed to be a general model to explain enacted behavior but on the other it is viewed as a specialized strengths-based action model to promote enacted behavior among minorities and the historically oppressed. This is because resilience which is also assessed with identity in IBT is particularly relevant when a fragile or highly stressed population is considered. Alternatively, resilience is pertinent when behaviors that defy and disempower social norms are enacted by anyone.

To introduce readers to IBT in this article, the relationship between identity and behavior is identified. This includes how identity guides behavior, along with other factors that assist with attainment of goal behaviors tied to identity. Regarding predicting behavior, take, for example, students who are preparing for school. A relationship exists between identification as a student and preparation and action to engage in education. At the beginning of the year, a student may shop for items, buy clothes, talk to classmates about what to buy, and so on. Many, or all, of these actions will relate to student identity; the level of interest in preparing for the year is related to identification as a future student. To examine this more closely, look at the behaviors displayed by youth who are most interested in starting school in the current climate. The degree to which they subscribe to a student identity as they make plans to begin the year is clear as they learn how to navigate remote learning and prepare to re-enter classrooms where they will interact with others face-to-face again.

Educators, researchers, and clinicians are encouraged to utilize and empirically test the efficacy of IBT with relevant samples to satisfactorily promote strength and resilience tied to identity among all individuals, including those who have been historically marginalized. This is done by learning about how to develop and use IBT questionnaires. Subsequently, IBT has been used to examine study skills among student athletes and emerging adults who are grieving, and, most recently, gender minority advocacy competence among 1,191 school counselors located throughout the United States (Simons, 2021). The identities that were assessed in the latter study in relation to behavioral enaction (advocacy acts for gender minority youth) were gender, sexual orientation, and race/ethnicity. School counselors who identified as transgender and gender non-binary were more likely to advocate for gender minority students than school counselors who identified as male or female. School counselors who identified as gay, lesbian, and mostly heterosexual were more likely to advocate for gender minority students than school counselors who identified as heterosexual. School counselors who identified as multiracial and European American were more likely to advocate for gender minority students than school counselors who identified as African American. These findings suggest that cisgender individuals, heterosexuals, and African Americans who are enrolled in school counselor training programs might benefit from more training about gender minorities and how to advocate most effectively for them. Moreover, it can be inferred from Simons’(2021) findings that it is plausible to utilize IBT to predict and promote state-dependent behaviors (Maltby et al., 2019). A host of other studies conducted over the past three decades also confirm this (Ajzen, 2011).

## Complexity of Self-Assessment

Our knowledge of identity assessment is limited. A myriad of identities (personal, social, etc.) exist and few recommendations have been made regarding how to assess and reflect over identity most effectively. This is disconcerting because according to Reicher (2004), the self is complex. For example, the presence of more than one salient identity (intersectionality) requires more innovative approaches to assessment (Abrams and Hogg, 2004). Meyer (2010) used both qualitative and non-dichotomous quantitative techniques to assess identity in a diverse sample of sexual minorities and found that racial/ethnic minorities found their sexual identities (LGB – lesbian, gay, and bisexual) to be equally important. Additionally, Latino and Black LGB individuals and White LGB individuals displayed resilience similarly (Frost and Meyer, 2008). Second, IBT is focused on oneself, which may include subscription to social norms that discount resilience (e.g., due to cultural differences) (Miller et al., 2017). The takeaway is this: identity is an individual construct that is also historical and cultural (Reicher, 2004). Suffice it to say that people may prefer to conform to social norms even though it means compromising their health, values, and identities as they work toward behavioral goals. Findings like this stemming from application of IBT to examining behavior in research studies, education, and clinical practice allow for further discussion about the prioritization of individuals’ needs. A variety of needs assessments are available to assist scholars, educators, and clinicians with navigating this territory (Drummond et al., 2019). Hornsey (2008) has pointed out that behaviors between different groups of people tend to differ based upon individuals’ perceptions of where they stand hierarchically regarding power and the likelihood of advancement, legitimacy, and stability. Future research should use IBT along with other measures to control for these variables. Last, IBT recognizes that most people benefit from positive connectedness while working toward behavioral goals. However, some people working successfully toward these goals may not seek out nor desire support.

### Identity, Behavior, and Motivation

A plethora of empirical research exists to support the idea that behavior is related to identity (Conner and Armitage, 1998; Hagger and Chatzisarantis, 2006; Rise et al., 2010). Additionally, society is ever changing and becoming more diverse; people work in positions where it is helpful for them to develop greater awareness about who they are in relation to others (Sugarman and Martin, 2018). This societal change began in the 1970s to promote more inclusiveness based on highlighting greater differences among people (Haslam, 2016). Since then support has been found for assessing identity in relation to promoting and predicting many actions such as health behaviors (Chatzisarantis et al., 2009; de Bruijn and van den Putte, 2012; Ries et al., 2012); food choices (Hagger et al., 2007; Dean et al., 2012; Caso et al., 2016; Carfora et al., 2019); academic behaviors (White et al., 2008, 2011; Pownall, 2012; Cheng and Chu, 2014); leadership (Steffens et al., 2014); and behaviors that impact the environment (Fielding et al., 2008; Nigbur et al., 2010; Whitmarsh and O’Neill, 2010; Reid et al., 2018; Peng et al., 2019).

**FIGURE 1:**
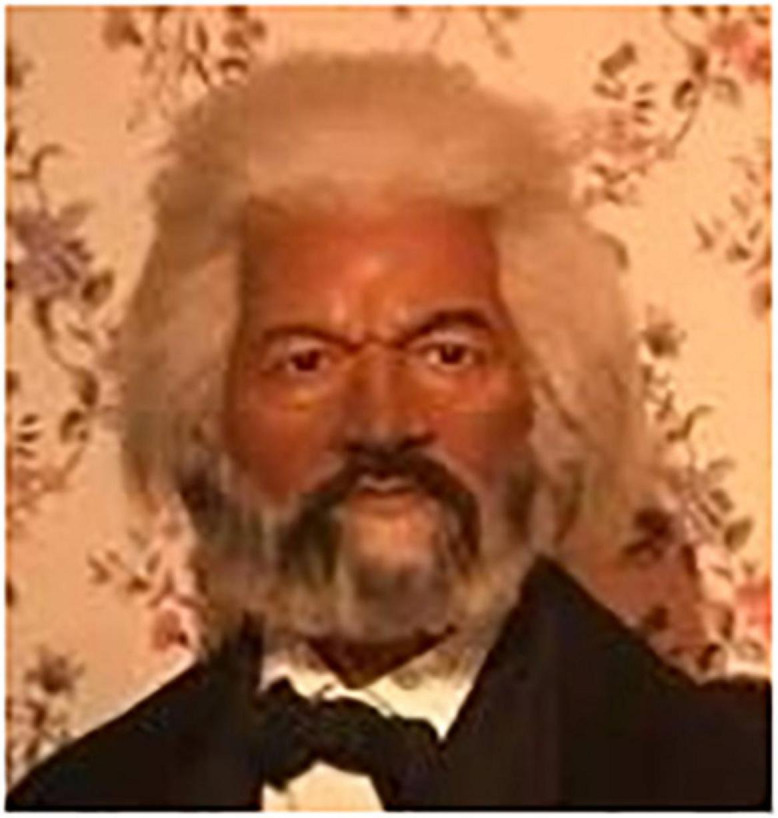
Likeness of Frederick Douglass. Wax figure representation in Washington, DC in 2020.

**FIGURE 2:**
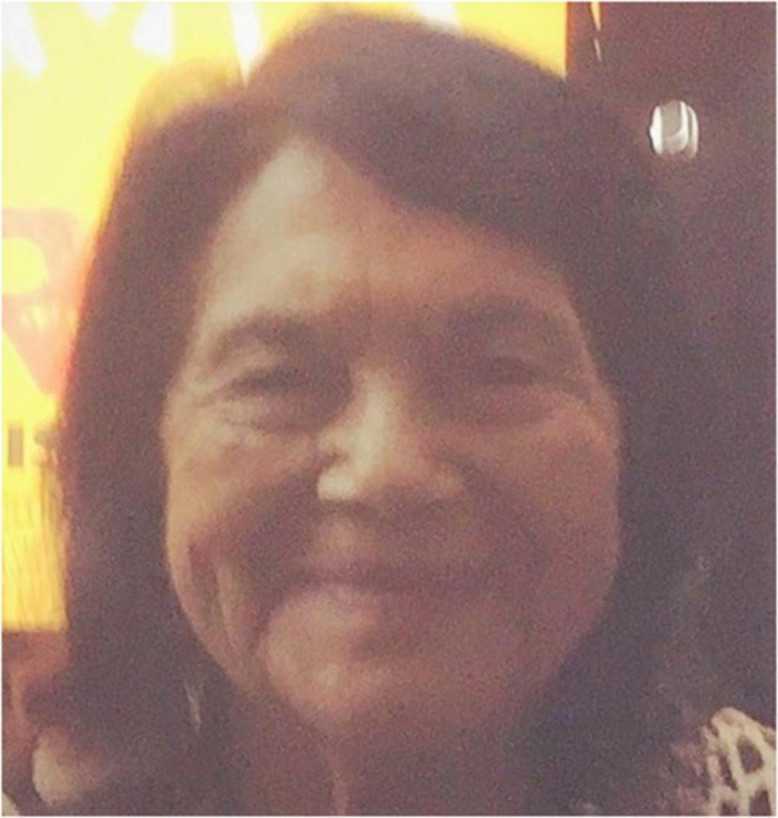
Dolores Huerta. Attending a premiere of a film about her life in New York City in 2017.

Another area related to the assessment of identity and behavior is motivation. IBT, as a strengths-based action theory, embraces aspects of motivational theories. For example, IBT embraces human motivation theory (MT; Maslow, 1943). IBT is concerned with the need to learn from individuals who have already successfully enacted goal behaviors. Concerning MT, these people include those such as Frederick Douglass (Figure 1), a former slave and social activist, and Dorlores Huerta (Figure 2), an American labor leader and civil rights activist. Maslow (1943) referred to them as exemplary individuals. In IBT, however, exemplary people like Douglass and Huerta are referred to as positive role models. Positive role models have successfully enacted behaviors that their admirers wish to enact. When “positive role models” are physically present or at least known of by individuals seeking to enact goal behavior, the role models’ true or symbolic presence serves as inspirational personal support. Further, IBT recognizes the need for individuals to ideally have all five hierarchical needs proposed by Maslow met, but this is not a requirement *per se*. MT does not explicitly address diversity and inequality (Wigfield and Koenka, 2020). Conversely, IBT does. The model recognizes that the human condition is diverse and that humans thrive in some aspects of life even though they might struggle in others.

This difference between the MT and IBT includes how each of the models deal with discrimination and racism, both of which influence motivation (Wigfield and Koenka, 2020), While IBT recognizes this, MT does not. IBT also recognizes that most individuals have the capacity to set and achieve behavioral goals tied to who they are, positive thoughts about themselves and their target behaviors, and recognition that receipt of positive support from others might be helpful to reach goals (Alemi et al., 2003). Maslow’s hierarchy of needs include physiological needs, safety, love and belonging, self-esteem, self-actualization, and self-transcendence. With IBT, only three of Maslow’s five hierarchical needs are highlighted: self-esteem, love, and physiological needs. Emphasis is placed on their role in the factor of resilience. In MT, Maslow viewed the role of physiological needs as more important than the role of self-esteem, but in IBT the role of self-esteem is viewed as more important than the roles of love and physiological needs, the latter of which are related to having the ability to regulate emotions in the face of challenge (Kahle et al., 2018).

## Identity Behavior Theory

**FIGURE 3:**
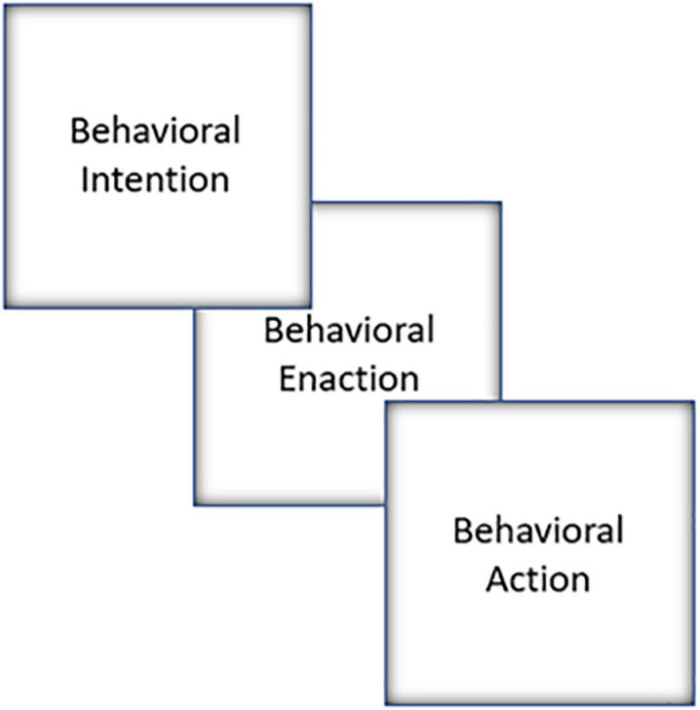
Identity Behavior Theory. Conceptual representation of Simons’ Identity Behavior Theory.

As aforementioned, IBT is a strengths-based action theory of learning and personal behavior. IBT recognizes diversity and minority stress. The model includes behavioral enaction, the process whereby individuals shape their experiences through actions (Rocha, 2012). IBT comprises attitudes, identity, resilience, and behavioral enaction (Figure 3). Building on Turner’s (2005) view of identity as a driver of personal strength, IBT emphasizes the importance of access to positive resources (Hornsey, 2008). IBT allows for testing new behavioral hypotheses, and, at its core, is concerned with how identity, along with attitudes and resilience increases the likelihood that one will try to, or successfully, enact behavior. IBT is focused on motivation, mindset, action, and support, each of which are essential to achieving behavioral goals. This stands in contrast with identity-based motivation (IBM) theory which emphasizes the impact of environment (Oyserman, 2015). IBM has been used to examine consumerism, and views contextual cues as more important for predicting and promoting behavior than individual differences and personal strength.

### Attitudes

Attitudes reflect how individuals have processed information that they have been exposed to. Attitudes serve as the cognitive foundation from which people respond to objects, and events (Ajzen and Cote, 2010). A review of the literature indicates that attitudes are not innate. A myriad of attitudes exists, including general and specific attitudes, and they are acquired over the course of one’s lifetime. Individuals may hold both strengths-based and deficit attitudes. Fortunately, however, new attitudes can be formed, and others’ attitudes may disappear or become in consequential. This is especially important to recognize in cases where individuals hold deficit attitudes and think less of themselves and their abilities because of their minoritized statuses or oppressed backgrounds. In IBT, attitudes are related to beliefs that one will successfully perform a specific task (Ajzen, 1991). Assessing and encouraging favorable specific attitudes toward target behaviors as part of behavior scales is useful in predicting and promoting behavior tied to identity (Ajzen, 1991, 2012). An example item is, “I hold a favorable attitude toward school.” The empirical examination of behaviors and attitudes together yield knowledge about applied areas such as leadership training, health behaviors, and advocacy (Terry et al., 2000). Concerning the latter, for example, Torrence (2012) studied the influence of school counselors’ attitudes on their ability to provide services to students with disabilities. One hundred and sixteen school counselors were surveyed. Positive attitudes toward students with disabilities were related to higher levels of ability to help. These findings suggested that not only were specific attitudes toward self and behavioral goals important, but specific attitudes toward others also were. In IBT, specific attitudes are assessed, not global attitudes. Decades of social psychological research has shown that unlike specific attitudes, global attitudes are too general to use in predicting behavior (Ajzen and Cote, 2010). Moreover, when specific attitudes have been assessed along with global attitudes, the influence of specific attitudes has consistently been found to be more relevant in assessing the likelihood of a behavioral outcome.

### Behavioral Enaction

According to Rocha (2012), “[behavioral] enaction is the notion that our worldly experience is created through the body shaped by our actions” (p. 4). To further define behavioral enaction, Hutchins (2010) shared:

[Behavioral] enaction is the idea that organisms create their own experience through their actions. Organisms are not passive receivers of input from the environment but are actors in the environment such that what they experience is shaped by how they act (….). Perception and action are not separate systems, but [they] are inextricably linked to each other and to cognition. This last idea is a near relative to the core idea of enaction. (p. 5)

Behavioral intention encompasses plans - made in advance – to perform a behavior. In IBT, behavioral intention is subsumed, along with behavioral action, into behavioral enaction. Behavioral intention includes predicted attempts (intra- or interpersonal) at planned behaviors and is assessed as part of behavioral enaction. Behavioral intention such as planning to act out a behavior may or may not lead to behavioral action. Nonetheless, both successful and unsuccessful behavioral performance is recognized as behavioral enaction in IBT, and points are awarded.

### Identity

**FIGURE 4:**
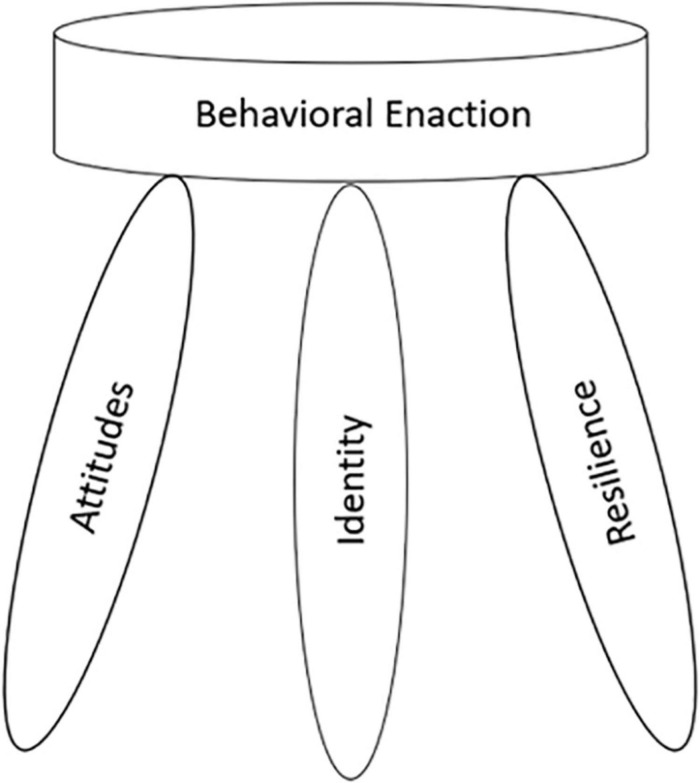
Behavioral enaction. Conceptual representation of the components of behavioral enaction by Simons.

Identity is an enduring and salient part of one’s internalized perception of self (Rise et al., 2010). These parts represent cognitive schemas and social categories which convey specific meaning(s) of behavior (Obschonka et al., 2015). An example is thinking about oneself in a way related to action (e.g., I am an athlete, so I work out) (Savickas, 2002). One’s identity as an academic is accounted for by having a sense of being part of the academy and viewing academia as part of oneself (Tajfel, 1972). Within IBT, identity determines if one can enact a behavior, or set of behaviors. One’s identity is central to behavioral performance. Foote (1951) argued that individuals without identities do not understand the makeup and behaviors of different groups of people. He also believed that these individuals were unlikely to meet behavioral goals and may resort to suicide in the absence of meaning in life tied to a salient identity. He wrote, “When doubt of identity creeps in, action is paralyzed. Only full commitment to one’s identity permits a full picture of motivation” (Foote, 1951, p. 18). Having a strong identity related to a task, positive attitudes toward the task, and a strong belief in doing the task (personal strength) increases the likelihood that one will commence or fully enact the task. Whereas the former “commenced outcome” is referred to as behavioral intention, the latter “successfully enacted outcome” is referred to as behavioral action (Figure 4). Behavioral intention and behavioral action are more alike than dissimilar, and both are assessed as part of behavioral enaction. Behavioral intention has often accounted for a significant amount variance in behavioral action (Ajzen, 2012). Much of this work on trying to predict behavior over the past three decades has focused on behavioral intention instead of identity (Ajzen, 2011). This has occurred despite the increasing amount of support to focus on the latter instead of the former when trying to predict and foster behavior.

### Resilience

Resilience is the ability of a person to adapt to stress and to recover from negative emotional experiences (Block and Kremen, 1996; Hu et al., 2015). The French Jewish psychiatrist, Boris Cyrulnik, studied resilience for over thirty years. Stemming from imprisonment in a Nazi concentration camp and loss of his parents as a child, he viewed resilience as an important psychological construct (Cyrulnik, 2009). He described it as a set of sturdy personality traits that could even be found in children who prospered despite having come of age in some of the fiercest environments (Cyrulnik, 2008). The definition of resilience in IBT draws on factors and items proposed by the authors to assess resilience tied to support and personal strength, including positive emotion regulation. Factors and items however that do not emphasize autonomy and strength are not included in the assessment of resilience. For example, a belief by others that one should engage in tasks that would undermine progress toward positive behavioral enaction (e.g., focus on negative affect or perception of stress in the past) would not be examined on an IBT questionnaire.

The factor of resilience supports three tenets of IBT: (a) not all identities are congruent with social norms (marginalized identities); (b) while some people try to follow social norms, others do or cannot; and (c) healthy behaviors are behaviors that are congruent with one’s identity (Simons and Beck, 2020). These behaviors are in harmony with the make up of people and how they view themselves. For example, one may subscribe to logic of appropriateness theory (LOAT; March, 1994). LOAT posits that people modify behavior to conform to the expectations of others. This, however, is not always the case because expectations of others might not be reasonable based on one’s identity and goals. Others’ expectations also change. Moreover, when people conform to the expectations of others to the detriment of their own identities, their mental wellness is often compromised over time (Simons and Beck, 2020). Individuals may eventually contemplate, attempt, or complete suicide (Foote, 1951; Simons and Beck, 2020). IBT, on the other hand, recognizes the importance of having support versus being pressed by others to behave in particular ways. While IBT emphasizes the role of human will over environmental factors, IBT does not discount the benefits that might be gained from interaction between individuals and their environment. To learn more about resilience beyond the scope of this article, refer to the readings in Table 1 and the Identity Behavior Theory Resilience Scale in Supplementary Appendix A. According to Maltby et al. (2019), over 25 resilience measures encompassing many constructs exist in the literature (Pangallo et al., 2015; Lock et al., 2020). Thus, no gold standard definition of resilience exists, and researchers continue to disagree if resilience is state-dependent, trait psychological, or a combination of both. Block and Block (1980) have come to view resilience as tied to personality traits, yet Lock et al. (2020) have come to view resilience as a state dependent construct. In IBT, resilience is viewed and assessed as the latter, as state-dependent, not as trait psychological. Like global attitudes, trait psychological resilience is too broad to be relevant in predicting target behaviors.

**TABLE 1:** Readings for further information on resilience.

**Authors**	**Areas of resilience**	**Highlights**
Block and Kremen, 1996; Letzring et al., 2005; Prince-Embry and Saklofske, 2013; Block, 2014	Resilience over the lifespan	Ego-resilience and longitudinal research
Cyrulnik, 2008, 2009; Oshio et al., 2018	Resilience during childhood and emerging adulthood	Personality trait characteristics
Dweck, 2016	Resilient mindset	Fixed versus growth mindset
Friborg et al., 2005; Friborg et al., 2017	Personal strength, family, and resources	Resilience Scale for Adults
Hu et al., 2015; Maltby et al., 2019	Trait resilience and ecological systems model of resilience	Mental health and Resilient Systems Scales
Lerner, 2006	Environment and person	Benefits of positive interaction
Lock et al., 2020	Assessment of resilience	State-dependent resilience
Schulz and Schwarzer, 2003	Emotional and instrumental support	Berlin Social Support Scales for Medical and Healthy Populations
Ungar, 2008, 2011	Culture and support	Adult Resilience Measure (ARM; Resilience Research Centre, 2018).

The assessment of resilience in IBT recognizes that enacting behavior requires having access to positive individual and group level resources which can be substituted for disempowering and deficit-based norms. Resilience comprises two factors: (a) support and (b) personal strength. Support comprises connection and resources. It also recognizes that people do not function in a vacuum. Most individuals benefit from having social support and being connected to others when trying to achieve their goals. The IBT paradigm therefore shows deference to helpful environmental forces (positive resources) that foster individual growth despite exposure to stress. Personal strength comprises agency, self-compassion, and emotional stability (emotional regulation). It is focused on the influence of one’s own views, physiology, and behavior and is related to how one responds behaviorally and emotionally when his, her, or their interests and goals are, or could be, thwarted. Agency is a belief in self that he, she, or they can sustain and enact behavior (Snyder et al., 1991). Emotional stability indicates that those who are more conscientious are less emotional (Friborg et al., 2005).

## Identity Theory

Identity theory posits that identity is one of the most influential psychological characteristics at the core of an individual (Haslam, 2014, 2016; Obschonka et al., 2015; Sugarman and Martin, 2018). Identity guides action in a variety of situations (Fauchart and Gruber, 2011). One’s self-concept comprises different role identities (e.g., employee, partner, and spouse) (Stryker and Burke, 2000). Identity comprises expectations of one’s social position. This is role-person merger, the degree to which one identifies *as* something (person, group of people, community, or organization). The extent to which a societal role is internalized within a person is reflected (Charng et al., 1988). Thoits and Virshup (1997) believed that identity exists to provide self-understanding at an individual level and consists of “ME” information. Identity also exists as interchangeable identification of oneself with a certain category or social group and consists of “WE” information (Thoits and Virshup, 1997). One identifies *with* something such as a person, group of people, community, or organization. IBT and identity theory are similar but different. Both theories assume that one’s behavior results from rational decision-making; however, identity theory is focused on interpersonal behavior and the broad social implications of behavior related to identity (Conner and Armitage, 1998; Stryker and Burke, 2000), whereas IBT explains both intra- and interpersonal behavior with more of a narrow focus on behavior influenced by identity.

Identity theory is related to LOAT (March, 1994). According to LOAT, individuals assess situations based upon their identities to discern how they should behave most appropriately (March, 1994). One’s identity is considered in a social space used to map out behavior (Case et al., 2016). LOAT is more aligned with social theory than identity theory, and thus places greater emphasis on the role of the social environment in influencing behavior. Behavior follows from institutionalized rules that govern an appropriate course of action. Alternatively, IBT focuses on one’s behavior tied to both identity and the social environment, with a caveat: IBT emphasizes the role of identity more than the role of the environment. Greater emphasis is placed on rational decision-making to enact behavior from an independent autonomous space with the ability to benefit from positive support from others, or helpful resources.

The inclusion of identity in IBT strengthens the paradigm. The idea that behavior is both independent and interdependent is reinforced. That is, behavior results not only from present decisions made in a vacuum but also from decisions made within a dynamic and interactive, social environment that may or may not be supportive (e.g., Charng et al., 1988). IBT recognizes the importance of assessing salient identities in conjunction with attitudes and resilience (Conner and Armitage, 1998). Last, the creation of IBT has been inspired by the Applied Social Identity Approach (ASIA; Haslam, 2014), which calls for understanding how social identities are related to individuals’ attitudes and behaviors. For example, political messages usually only affect people when those people possess the same identities of those who have created the messages (Tarrant et al., 2011). As a result, some attempts to influence people are futile or go awry (Haslam, 2014).

## Identity in Behavioral Intention and Enaction

Two decades ago, Conner and Armitage (1998) recommended assessing identity and five other variables: affective beliefs, belief salience, moral norms, past behavior, and self-efficacy, the latter of which differs from attitudes. Self-efficacy is assessed separately from attitudes in IBT and is concerned with the likelihood of being able to enact goal behavior. Attitudes, however, are more concerned about the qualities of behavior, not the ability to enact it or not. Of these variables, identity has received the most support in the literature over time for its use along with other factors to predict behavior. Rise et al. (2010) conducted a meta-analytic study to examine identity. Forty independent tests (*N* = 11,607) were examined, and an average correlation of 0.47 across tests was found between identity and behavior. Identity accounted for six to nine percent of the variance in behavioral intention. de Bruijn and van den Putte (2012) studied exercise identity among 538 undergraduates and the factor was found to be the strongest predictor of exercising. Further, levels of behavioral intention (plans to exercise) among students, along with exercise identity were at least triple that of levels of behavioral intention to exercise among students without exercise identity. White et al. (2011) surveyed 46 undergraduates to examine identity regarding attendance at voluntary statistics review sessions. Both self- and group identity predicted attending scheduled review sessions; however, as the semester progressed, self-identity became a better predictor of attendance than group identity.

While a thorough assessment of the underlying mechanisms of behavior requires examining each of the IBT constructs in relation to each other, researchers may choose to assess a fewer number of IBT relationships, and thus ask fewer research questions. In some cases, the relationship between identity and behavioral enaction may be significant. However, in other cases, it may not be. Concerning this outcome, assessing all IBT constructs (attitudes, resilience, identity, and behavioral enaction) together is recommended. When a strong relationship between identity and behavior is hypothesized though, identity may need to be further examined. Scholars are encouraged to develop and refine non-dichotomous identity scales on IBT measures (Meyer, 2010). This allows them to measure identity of respondents without imposing or forcing identity conflict as part of the response process. Along with survey items, identity scales should also have a short answer text box that can be used by respondents to share more about how they view and subscribe to identity or a set of identities. Collection of these data is especially helpful because they illuminate qualitative findings and allow respondents to elaborate more in depth about identity, identity development, and more complex identities such as those that involve intersectionality.

## Implications

Identity Behavior Theory can be integrated into a variety of paradigms including, but not limited to, social justice theory, existential and feminist paradigms, and cognitive-behavioral and psychodynamic paradigms. Cognitive-behavioral paradigms that work well with IBT (e.g., to improve leadership skills) include behavioral, cognitive behavioral, and rational emotive behavioral theories. Psychodynamic paradigms that work well with IBT include psychoanalytic, analytic, and Adlerian theories. This is also the case with social justice counseling because IBT recognizes the effects of minority stress and inequality.

Identity Behavior Theory encourages people to utilize “I am; therefore, I do” cognitive behavioral exercises that are developed from IBT questionnaires. Item statements contribute to the development of positive self-talk, thought records, and daily affirmations. Individuals use IBT exercises to think more rationally (Edner and Cassiello-Robbins, 2020). IBT, for example, may be used to help employees better understand work expectations (van Dick and Haslam, 2012), or to improve their sense of self as a minoritized or oppressed person The paradigm may also help individuals to work with their employers to develop mutual understanding about how individuals’ identities are related to work skills, health, program development, and policies. The interventions are needed because in the absence of clear work policies, employees have reported experiencing reduced motivation and engagement (Foote, 1951; Haslam et al., 2020).

### IBT Questionnaire

The development of IBT questionnaires for use in predicting and promoting behaviors (e.g., positive health behaviors) are clear and well-founded in the literature. To examine all IBT relationships that might account for behavioral enaction, educators, researchers, and clinicians must learn to develop and utilize IBT questionnaires to examine goal behaviors. See Supplementary Appendix B for an abridged version of an IBT questionnaire with these scales created to examine one’s progress toward trying to work on homework at a desk for one hour uninterrupted several times a week over the semester. The abridged IBT questionnaire comprises the IBT Attitudes Subscale (IBT-A), the IBT Identity Subscale (IBT-I), the IBT Resilience Subscale (IBT-R), and the IBT Behavioral Enaction Subscale (IBT-BE). When developing an IBT questionnaire (a) a pool of items is developed from a comprehensive literature review with input from a panel of experts and (b) the experts review each of the scale items and assign content validity ratings to each item (0 = *Not necessary*, 1 = *Useful but not essential*, or 2 = *Essential*). Items with average content validity ratings less than two are removed from the measure. As a strengths-based action measure, all items on IBT questionnaires are positively worded and are not reversed scored. When creating an IBT questionnaire, inflammatory language should not be used.

### Identity Subscale Development

Although a paucity of research exists on how to effectively assess identity, several ways have been recommended (Burke, 1980; Terry et al., 1999; Meyer and Ouellette, 2009; Meyer, 2010; Oyserman, 2015; Simons et al., 2017). According to Terry et al. (1999), an identity subscale has two items to assess the level of enactment or non-enactment of behavior tied to identity. Example items include (1) “Being a student is an important aspect of who I am,” and (2) “I am not oriented to being a student.” The items are scored on a four-point Likert-type scale with values ranging from (1) *strongly disagree* to (4) *strongly agree*. The second item is reverse scored. A total score is calculated by adding the two scores together; higher scores indicate greater subscription to student identity. Lower scores indicate lesser subscription to student identity. Meyer and Ouellette (2009) have recommended using both qualitative and non-dichotomous quantitative measures to assess identity. According to Meyer (2010), this helps one to learn about the complexity and subtle differences among identities, prevents suppression of identities by other identities, and allows individuals to subscribe to more than one identity.

An identity subscale may also be created from an identity definition found in the literature. Simons et al. (2017) surveyed 392 school counselors about advocacy activity in relation to social justice advocacy identity. Social justice advocacy was defined as a political process in which counselors faced challenges, picked sides, self-promoted, built intentional relationships, and taught students advocacy skills (Singh et al., 2010). The school counselors rated this advocacy identity definition as it applied to them on a six-point Likert-type scale ranging from (1) *not at all true* to (6) *totally true*. Higher scores indicated that they self-identified as social justice advocates based on the advocacy definition and practiced advocacy activities more. Lower scores indicated that school counselors self-identified as social justice advocates based on the advocacy definition and practiced advocacy activities less. Next, Oyserman (2015) has suggested assessing how congruent or incongruent one’s behavior is in relation to goal behavior as part of her IBM model. More research certainly is warranted in this area because, unlike IBM, IBT does not consider how congruent or incongruent behavior is *per se*; IBT is most concerned with the degree to which one subscribes to identity in relation to congruent behavior, not the degree to which one has comfortably attained an identity, or what others think about regarding one’s identity. Moreover, IBT, emphasizes studying the identity-to-congruent behavior linkage noting that stronger identities are more likely to support this linkage than weaker identities. Last, Burke (1980) has recommended standardizing identity measures to (a) assess more than one identity; (b) connect identity to one’s life roles; (c) assess counter-identities (e.g., teacher versus student); and (d) assess how individuals are motivated by identities. Specific recommendations for how to do this though remain unclear, but they hold promise for assessing multiple identities.

## Discussion and Future Research

To support scholarship in this area, researchers should conduct well-designed studies (e.g., factorial design studies) to test IBT in predicting a variety of behaviors among different groups of people, especially those with marginalized and invisible identities. For example, an IBT questionnaire could be administered to assess academic skills, health behaviors, and food choices with first generation college students, immigrants, LGBT students, racial minorities, or females in STEM. Experimental studies that go beyond assessing relationships between longitudinal measures (e.g., lifespan measures) should also be conducted (Weinstein, 2007). Overall, at a minimum, it would be promising to show the results of a pretest of the IBT questionnaire on a relevant sample and analyze whether the full IBT model can be satisfactorily tested empirically using an IBT questionnaire. According to Noar and Head (2014):

Behavioural theories are taught and applied across multiple disciplines. They are used as a basis for many intervention types, including behavioural interventions, eHealth applications and mass media campaigns. They are used in cancer, HIV/AIDS, obesity and tobacco, among numerous other areas. They underlie scores of research studies funded by external funding agencies. And they are continually taught to the next generation of health behaviour researchers, who we hope can take even greater steps forward than those that came before them. (p. 68)

Optimistic IBT will predict and encourage successful behavioral enaction among a wide array of individuals, researchers should develop research questions to examine the application of IBT to assess the potential of individuals to enact new behaviors in applied settings, including schools, and research labs. Because we do not just want to say that certain identities lead to certain behaviors, we need to be clear about what is contributing most to behavioral outcomes. Developing and testing hypotheses with representative data and examining identity predictors as part of a central study based on larger, representative samples is recommended to yield valuable knowledge. For example, the resilience hypothesis could be tested among different groups to identify which individual- and group-level resources are most helpful to those who experience societal prejudice (Meyer, 2015). Further, a review of the literature suggests that testing attitudes, identity, and resilience as predictors of target behaviors—independent of each other and altogether—is warranted (Ajzen, 2011, 2012). The factors of resilience and identity should also be tested as mediators and moderators with respect to enacted behavior. IBT is integrative, and support has been found for testing identity (e.g., age, gender, degree of compartmentalization, etc.) and resilience (e.g., hardiness, social support, and coping) as mediators and moderators in studies with transgender individuals seeking better health (Komosa-Hawkins and Schanding, 2013; Etengoff and Rodriguez, 2020); Black White biracial Christians experiencing discrimination (Vazquez et al., 2021); hotel managers engaging with employees (Buil et al., 2019); nursing students who feel alienated (Bekhet et al., 2011); university students who smoke or who are at risk of dropping out (Robinson, 2003; Sun et al., 2011); individuals experiencing chronic pain (Dima et al., 2013); and adolescents affected by depression (Ng and Hurry, 2011).

Using IBT, researchers should also examine new forms of advocacy for self and others in healthcare and education, as well as develop new IBT interventions (identity behavior therapy). As aforementioned, social identities meet basic psychological needs: the need to belong, the need for control, and the need for meaningful existence (Maslow, 1943; Greenaway et al., 2015). As such, IBT applications can increase academic success, health, and wellness. This outcome is novel in that the founders of social identity theories largely overlooked establishing protocols to apply their theories outside of social psychology (Haslam, 2014). With IBT, attention is given to empowering others, especially those who have been historically marginalized. The French West Indian psychiatrist Frantz Fanon believed that a viable identity was key to bringing about positive development (Fanon, 1963). He believed that compartmentalization of racial identity served as an example of one of the ill effects of colonialism (Fanon, 1963). This outcome had a negative cascading effect; of those who compartmentalized their identities, many lacked resilience, experienced burnout, tried to commit suicide, and, in general, had poorer mental health when compared to those who had not compartmentalized their identities (Foote, 1951; Morrow et al., 2018; Meyer et al., 2021). These effects of minority stress still occur today (Moule, 2009). As a result, IBT training programs and counseling interventions should aim to develop more language and knowledge around comfortable autonomy, other demographic variables, and attitudes and resilience in relation to degree of subscription to identity and goal (future) behavior (Albarracin et al., 2001; Sandberg and Conner, 2008; Rise et al., 2010; Kor and Mullan, 2011; Simons and Beck, 2020).

Researchers have also recommended studying personality tied to behavior (Foote, 1951; Cyrulnik, 2008, 2009; Hu et al., 2015; Maltby et al., 2019; Lock et al., 2020). Some have recommended studying extraversion and introversion noting that those who identify as extraverts tend to be more resilient than those who identify as introverts (Allport, 1937; Crum et al., 2013; Livingston et al., 2015). Additionally, by testing the IBT along with measuring personality traits, the theory and IBT questionnaire may be further refined and validated to predict and promote both state-dependent resilience (Maltby et al., 2019). The IBT factors may be assessed in relation to each other and personality traits while controlling for social desirability. To do this, either an abridged or unabridged version of an IBT questionnaire would be administered along with the Self-Monitoring Scale (SMS; Klar, 2021) and one or more personality measures such as the Big Five Personality Measure (Rammstedt and John, 2007; Oshio et al., 2018). Much of the scholarship on the construct of resilience over the past two decades has been conducted with minority populations. This seems like a plausible approach given the importance of resiliency among minoritized groups. However, because of this, scholars have neglected to learn more about resilience using samples from majority culture. As such, this is another area to be explored. Like learning about resilience from a minority worldview, learning about resilience from a majority worldview also helps us improve our understanding.

Future research with IBT should also include examination of the IBT factors, along with mindset which is related to resilience (Dweck and Yaeger, 2019). Mindset is about one’s beliefs/thoughts about overcoming a challenge (Dweck, 2016). If something is challenging, one says, “I’ll never get this, I am no good at biology,” or one says, “I just need some more time in the lab, and I think I can figure it out.” In essence, the challenge is either perceived as a barrier that inhibits progress or is perceived as an obstacle that needs to be navigated around using effort. In these instances, IBT experts ask, “Are participants talking about exhibiting consistent effort over time? If so, this would constitute mindset” (R. Trenz, personal communication, January 26, 2021). Mindset has been assessed to predict how one will likely respond to encountering stress (Crum et al., 2013). According to Dweck (2016), two types of mindsets exist: (a) fixed and (b) growth. A fixed mindset is the belief that qualities are carved in stone, and success comes from innate ability. One says, “I am just not good at math. I will never be able to pass calculus.” Growth mindset is the belief that qualities are things that can be cultivated through efforts, strategy, or help from others, and success comes from hard work and persistence. One says, “Math is not my strongest subject, but I will be able to get through calculus by working hard and getting help from a peer tutor.” According to Dweck (2016), holding a growth mindset is more favorable than holding a fixed mindset.

## Limitations

Some critics of IBT and use of IBT questionnaires disfavor using empirical research to assess behavior because they believe that findings from aggregate data lack generalizability, and that causal inquiry is not appropriate for examining human behavior (Barrow and Foreman-Peck, 2005; Power, 2014). Nonetheless, findings from aggregate data can be used to predict patterns in group behavior (Martin, 2019). Other critics of IBT do not see the value in recognizing and encouraging acceptance of self and individual differences to promote inclusion (Haslam, 2016). Sugarman and Martin (2018) suggested that when inclusion is promoted by way of differences, it fosters victimhood, lack of civil discourse, and the need for therapeutic services. These outcomes, however, run contrary to the tenets of IBT, which are exactly the opposite. The three tenets of IBT support an additional tenet: people have inherent worth, and they benefit from individuality and independence, and acting based on their strengths, instead of their deficits, is noble and of value. Another concern is support for IBT in this article based mostly on a comprehensive literature review and not on self-collected empirical data using IBT questionnaires. Therefore, the empirical methodology cannot be evaluated. However, the inferences drawn from the reviewed works on identity or resilience are substantive and reasonable.

## Conclusion

We have presented Identify Behavior Theory (IBT) and have described how it may be used to examine and promote behavioral enaction. Yet, despite long-standing support for the inclusion of identity in examining behavior for a variety of reasons (Rise et al., 2010; Ajzen, 2012), some debate the need for this paradigm. Nonetheless, it is extremely difficult to think about behavioral enaction in the world without naming and reflecting on the core IBT constructs: attitudes, resilience, and, most notably, identity. Those who find it challenging or fascinating to examine their own behaviors, and the behavior of others, in relation to identity appear likely to utilize IBT in their work and hold values that reflect the core tenets of IBT. The four tenets of IBT are (a) not all identities are congruent with social norms; (b) some people cannot or will not follow social norms; (c) healthy behaviors are congruent with identity; and (d) every person has value and benefits from being independent and striving for individuality to enact behaviors. Hornsey (2008) wrote “many of the meta-theoretical and ideological wars that were waged by social identity theorists have been largely won” (p. 217). In IBT self-identity is considered and promoted as a motor for action. Today, the emerging use of the IBT paradigm in research, education, and clinical practice holds the potential to advance our knowledge and understanding of identity and how it can be more effectively assessed to improve the lives of everyone.

## Data Availability Statement

The original contributions presented in the study are included in the article/Supplementary Material, further inquiries can be directed to the corresponding author/s.

## Author Contributions

The author confirms being the sole contributor of this work and has approved it for publication.

## Conflict of Interest

The author declares that the research was conducted in the absence of any commercial or financial relationships that could be construed as a potential conflict of interest.

## Publisher’s Note

All claims expressed in this article are solely those of the authors and do not necessarily represent those of their affiliated organizations, or those of the publisher, the editors and the reviewers. Any product that may be evaluated in this article, or claim that may be made by its manufacturer, is not guaranteed or endorsed by the publisher.
