# Supplementary material for: From Identity to Enaction: Identity Behavior Theory
Source: Front Psychol. 2021 Aug 24;12:679490. doi: 10.3389/fpsyg.2021.679490 (PMC8423104; doi:10.3389/fpsyg.2021.679490)
Supplement: Supplementary file 1 [file Data_Sheet_1.pdf]

Appendix A

Example: Identity Behavior Theory Resilience Scale (IBT-R)

To develop a resilience scale, it is recommended to use the following items and write the target behavior into the blanks. An IBT resilience scale comprises a personal strength subscale and a support subscale. Examples of personal strength include self-compassion, self-esteem, emotional stability, agency, and mindset. Examples of support include connecting with others (family, friends, partners, etc.), having access to resources, and wanting to have support.

Using the following scale, rate each item by selecting the appropriate number.

_____ 1 Not at All True
_____ 2 Mostly Not at All True
_____ 3 Somewhat Not at All True
_____ 4 Somewhat True
_____ 5 Mostly True
_____ 6 Totally True

Personal Strength Subscale

1. I am curious about and interested in ___________________.
2. It is important to me to ___________________.
3. I am committed to doing tasks that will move me closer to ___________________.
4. It is my responsibility to ___________________.
5. When I succeed at trying to ___________________, I am okay with the outcome.
6. With trying to achieve my goal to ___________________, I am patient and understanding towards myself.
7. If trying to ___________________ causes me pain, I will try to take a balanced view of the situation.
8. If I am unsuccessful at ___________________, I will see my failure as part of being human.
9. If I am unsuccessful at ___________________ and it makes me feel inadequate, I will remind myself that feelings of inadequacy are shared by most people.
10. Right now, I see myself as likely to be able to ___________________.
11. I have enough energy to ___________________.
12. I am consistently preparing myself to ___________________.
13. When I want to ___________________, I have no problem trying to.
14. I will likely succeed at ___________________.
15. I will think carefully about ___________________ before I do it.
16. My strong personality helps me ___________________.
17. My goal to ___________________ is possible to accomplish.
18. I am at my best when I ___________________.
19. If trying to ___________________ becomes upsetting, I will keep my emotions in balance.
20. I am cheerful because I am trying to ___________________.
21. Regarding trying to ___________________,
    21a. I feel calm, relaxed, or easy going.
    21b. I can focus.
    21c. I am confident that something good might happen.
    21d. If I can do it, I will feel useful.

Support Subscale

When “others” are referred to, these are significant referents. These individuals include, but are not limited to, parents, partner(s), family members, and friends.

1. I feel valued and important enough by others to try to ___________________.
2. I feel cared for enough by others to try to ___________________.
3. I have others in my life who like to spend time with me when I am trying to ___________________.
4. There are others in my life who will stand by me if I am not successful at trying to ___________________.
5. I am treated fairly by those who also try to ___________________.
6. I do not have difficulty telling others about my behavioral goal of ___________________.
7. I am comfortable trying to ___________________ with my significant others around.
8. Others in my life will support me in my desire to ___________________.
9. I can discuss my goal to ___________________ with others who I am close to in my life.
10. Other people in my life appreciate my desire to ___________________.
11. Others stand by me even if trying to ___________________ becomes difficult.
12. I feel supported and encouraged by others to ___________________.
13. The effort I put forth to ___________________ would be honestly rated highly by others.
14. When needed, I always have someone who can help me try to ___________________.
15. When I try to ___________________, other people show me that they like me.
16. When I try to ___________________, there are people there to always help me.
17. Before I try to ___________________, I will need to ask others for their advice.
18. If I fail at ___________________, I will look for someone to cheer me up.
19. If I need help to ___________________, I will ask others for it.
20. I have had or seen role models who also try to ___________________.
21. If I succeed at ___________________, I will feel connected to others who celebrate my success with me.
22. People like me when I ___________________.
23. Regarding my goal to ___________________, I have others in my life who:
    - 23a. Love and accept me.
    - 23b. Comfort me.
    - 23c. Encourage me not to give up.
    - 23d. Are there when I need them.
    - 23e. Take care of things I cannot manage on my own.
    - 23f. Help me find something positive in my situation.
