# Supplementary material for: From Identity to Enaction: Identity Behavior Theory
Source: Front Psychol. 2021 Aug 24;12:679490. doi: 10.3389/fpsyg.2021.679490 (PMC8423104; doi:10.3389/fpsyg.2021.679490)
Supplement: Supplementary file 2 [file Data_Sheet_2.pdf]

*Appendix B*

**Example: Abridged Identity Behavior Theory Questionnaire**

Unless otherwise indicated, use the following scale to rate each item by selecting the appropriate number.

_____ 1 Not at All True
_____ 2 Mostly Not at All True
_____ 3 Somewhat Not at All True
_____ 4 Somewhat True
_____ 5 Mostly True
_____ 6 Totally True

**Example: IBT Attitudes Subscale (IBT-A)**

1. It would be good for me to work on homework at a desk for one hour uninterrupted several times a week over the semester.
2. It is pleasant to work on homework at a desk for one hour uninterrupted several times a week over the semester.
3. I hold a positive attitude toward working on homework at a desk for one hour uninterrupted several times a week over the semester.
4. I hold a positive attitude toward others who have the same goal to work on homework at a desk for one hour uninterrupted several times a week over the semester.

**Example: IBT Identity Subscale (IBT-I)**

1. I am proud to display my student identity outwardly as one who is trying to work on my homework at a desk for one hour uninterrupted several times a week over the semester.
2. I am proud to be a student who is trying to work on homework at a desk for one hour uninterrupted several times a week over the semester.
3. I feel connected to other students who are trying to work on homework at a desk for one hour uninterrupted several times a week over the semester.
4. I like myself as a student who is trying to work on my homework at a desk for one hour uninterrupted several times a week over the semester.
5. My identity will be affirmed when I work on my homework at a desk for one hour uninterrupted several times a week over the semester.
6. I identify as a student who is trying to work on homework at a desk for one hour uninterrupted several times a week over the semester.
7. What else would you like to share about concerning your identity tied to working on homework at a desk for one hour uninterrupted several times a week over the semester? (fill in the blank)

**Example: IBT Resilience Subscale (IBT-R)**

1. I am curious about and interested in trying to work on my homework at a desk for one hour uninterrupted several times a week over the semester.
2. I am treated fairly by those who also try to work on homework at a desk for one hour uninterrupted several times a week over the semester.
3. Other people in my life appreciate my desire to work on my homework at a desk for one hour uninterrupted several times a week over the semester.
4. When I try to work on homework at a desk for one hour uninterrupted several times a week over the semester, other people show me that they like me.
5. If I fail at trying to work on my homework at a desk for one hour uninterrupted several times a week over the semester, I will look for someone to cheer me up.
6. I feel valued and important enough by others to work on homework at a desk for one hour uninterrupted several times a week over the semester.
7. Regarding my goal to work on homework at a desk for one hour uninterrupted several times a week over the semester, I have others in my life who love and accept me.

**Example: IBT Behavioral Enaction Subscale (IBT-BE)**

Pertaining to your goal to work on homework at a desk for one hour uninterrupted several times a week over the semester, please rate each item as it applies to you by selecting the appropriate number. Choose only <u>one</u> response for each item.

_____ 0 I have not enacted this activity.
_____ 1 I made plans to enact this activity in advance, but I did not enact it.
_____ 2 I tried to enact this activity but was unsuccessful in doing so.
_____ 3 I successfully enacted this activity.

1. I have a desk of my own to work on doing my homework one hour uninterrupted several times a week over the semester.
2. I participate in trainings to become better at working on my homework at a desk for one hour uninterrupted several times a week over the semester.
3. I tell others about my goal to work on my homework at a desk for one hour uninterrupted several times a week over the semester.
4. I maintain contact information for local resources to help me work on my homework at a desk for one hour uninterrupted several times a week over the semester.
5. I work with others to develop the skills to work on my homework at a desk for one hour uninterrupted several times a week over the semester.
6. I work alone on my homework at a desk for one hour uninterrupted several times a week over the semester.
7. I select the assignments in advance that I will do for my homework at a desk for one hour uninterrupted several times a week over the semester.
